# The Occurrence and Outcomes of Cemento-Osseous Dysplasias (COD) in the Jaw Bones of the Population of Lower Silesia, Poland

**DOI:** 10.3390/jcm13226931

**Published:** 2024-11-18

**Authors:** Kamil Nelke, Jacek Matys, Maciej Janeczek, Agata Małyszek, Klaudiusz Łuczak, Marceli Łukaszewski, Marta Frydrych, Michał Kulus, Paweł Dąbrowski, Jan Nienartowicz, Irma Maag, Wojciech Pawlak, Maciej Dobrzyński

**Affiliations:** 1 Maxillo-Facial Surgery Ward, EMC Hospital, Pilczycka 144, 54-144 Wrocław, Poland; klaudiuszluczak@gmail.com (K.Ł.); dr.irmamaag@icloud.com (I.M.); wojciech.pawlak.mfs@gmail.com (W.P.); 2 Academy of Applied Sciences, Health Department, Academy of Silesius in Wałbrzych, Zamkowa 4, 58-300 Wałbrzych, Poland; 3 Dental Surgery Department, Wroclaw Medical University, Krakowska 26, 50-425 Wroclaw, Poland; 4 Department of Biostructure and Animal Physiology, Wrocław University of Environmental and Life Sciences, Kożuchowska 1, 51-631 Wrocław, Poland; maciej.janeczek@upwr.edu.pl (M.J.); agata.malyszek@upwr.edu.pl (A.M.); 5 Department of Anaesthesiology and Intensive Care, Sokołowski Hospital, Sokołowskiego 4, 58-309 Wałbrzych, Poland; marceliluk@gmail.com (M.Ł.); frydrych.marta.k@gmail.com (M.F.); 6 Division of Ultrastructural Research, Wrocław Medical University, 50-367 Wrocław, Poland; michal.kulus@umw.edu.pl; 7 Division of Anatomy, Department of Human Morphology and Embryology, Wrocław Medical University, 50-367 Wrocław, Poland; pawel.dabrowski@umw.edu.pl; 8 Private Practice of Maxillo-Facial Surgery, Romualda Mielczarskiego 1, 51-663 Wrocław, Poland; nienartowicz@gmail.com; 9 Department of Pediatric Dentistry and Preclinical Dentistry, Medical University in Wrocław, Krakowska 26, 50-425 Wrocław, Poland; maciej.dobrzynski@umw.edu.pl

**Keywords:** cemento-osseous dysplasia, jaw bones, mandible, periapical lesion, fibro-osseous lesion

## Abstract

**Background:** Cemento-osseous dysplasias (CODs) are rare lesions of the jawbone. Their occurrence, localization, type, size, and shape can vary between cases. This fibro-osseous lesion is typically found in the jaw near tooth-bearing areas and is often asymptomatic, discovered incidentally, and may be associated with the periapical region of the teeth. In rare cases, COD can lead to secondary bone osteomyelitis. Currently, there is limited information in the literature on the occurrence and characteristics of COD. This paper’s main aim was to focus on the authors’ COD experience in the lower Silesian area. **Methods:** A retrospective evaluation of radiographies (RTG-Panx, cone-beam computed tomography (CBCT)) was conducted on patients treated, diagnosed, or consulted by the authors. A statistical correlation analysis was made to establish any relationship within the gathered data. **Results:** COD is predominantly an incidental finding in the mandibular bone near tooth apices. It is most commonly diagnosed in females. Both CBCT and panoramic radiographies are generally sufficient for diagnosing the lesion. COD rarely requires treatment. **Conclusions:** COD lesions are mostly discovered incidentally during routine radiographies or cone-beam computed tomography (CBCT) scans. In most cases, clinical and radiological monitoring is sufficient, along with evaluating the teeth’s response to cold stimuli and assessing the surrounding bone structures. Biopsies or tooth extractions are seldom necessary. When oral hygiene is well-maintained and no periapical inflammation is present, COD lesions typically remain asymptomatic.

## 1. Introduction

The jaw bones can manifest various types of benign and malignant bone conditions. In terms of radiological appearance, they can manifest as radiolucent, radiopaque, or as mixed conditions. Furthermore, they can manifest in various shapes and sizes with both clinical and radiological appearances, like cystic, solid, or a combination of both, associated with odontogenic or non-odontogenic origins [1]. Both odontogenic cysts and tumors are well-known and presented in recent studies [2]. Their occurrence is mostly related to intraosseous features, quite often near tooth-adjacent or tooth-bearing structures. In some cases, extraosseous lesions, soft tissue involvement, and other manifestations can be found. Both solid, cystic, or other lesions appear in routine radiographies, and CBCT (cone-beam computed tomography) studies are well-described in the literature. Radiopaque lesions include fibrous dysplasia (FD), odontoma and odontoma-like lesions, idiopathic sclerotic lesions (also known as dense bone islands, DBI), dysplastic bones, osteomas, Paget’s disease, or others with both odontogenic or non-odontogenic origins [3,4].

A detailed radiologic evaluation is necessary to estimate the possible features and diagnoses of the evaluated lesion in the jaw bone. Cystic lesions can be quite easily investigated, especially when adjacent teeth are close to the lesion. The scope of symptomatology and varied possibilities of different pathologies manifesting as cysts require not only detailed CBCT evaluation but also sometimes a diagnostic biopsy. According to Holmes et al., the radiopaque lesions could be described as densely sclerotic (odontoma, COD-cemento osseous dysplasia, or osteoma), ground glass (COF—cemento-ossyfing fibroma, FD or Paget’s Disease), or mixed lytic–sclerotic (developing odontoma or CEOT—calcifying epithelial odontogenic tumor) in the radiographies [5,6,7]. Many fibro-osseous and bone lesions within the jaw bone have quite similar histopathological similarities; therefore, a good radiological and clinical evaluation might influence the final diagnosis. Furthermore, each lesion should be compared to the patient’s past radiographies/computed tomography images, or additional RTG/CBCT/CT evaluation should be scheduled in a 3–6 time frame to improve the diagnosis and decrease any diagnostic limitations.

Among all jaw bone lesions, cemento-osseous dysplasias (CODs) are quite an interesting. They are non-neoplastic asymptomatic lesions manifesting as fibro-osseous lesions with varying degrees of mineralization within close proximity to the teeth, dental arches, and adjacent tooth-bearing structures. Quite commonly CODs are more commonly found in the mandible. Over the years, their classification has changed, and, currently, they are a non-neoplastic bone lesion and can be seen in focal, periapical, florid, and familial forms [3,4,5].

Most commonly, single lesions are found; however, multiple CODs are not rare findings. COD usually manifests as an intraosseous lesion, sometimes expanding to the edge or external cortical bone layer. Early COD formation might mimic periapical granulomas, cyst formation, idiopathic osteosclerosis, chronic sclerosing osteomyelitis, odontoma, cemento-ossyfing fibroma, cementoma, osteoma, or similar bone lesions. Over time and their maturation, their final shape and self-limiting appearance grow. It is quite important to understand that COD microscopic evaluation is insufficient; because of its bony origin and fibro-osseous characteristics, it is impossible to differentiate its etiology, and, furthermore, this lesion has more than similar or even identical histopathological features to some other bone lesions [4,5,6,7,8]. Therefore, clinical and radiographic images are the most important diagnostic tools.

Rarely, some authors have reported an expansive, progressive type, which might suggest also that some similar fibro-osseous lesions might be familiar to classical COD. Some authors identify COD manifestation as a mixed lesion, consisting of woven bone and cementum-like features with a characteristic “ginger-root” pattern. This is mostly related to its form, composed of fibro-osseous structures with various stages of sclerotic mass inside [5,6,7,8,9,10]. Because of this, COD might have a reduced radiological appearance, namely complete radiolucent or mixed radiolucent–radiopaque with a radiolucent rim with more or less sclerotic lesions inside.

CODs can be found as solitary, multiple/multifocal lesions in tooth-bearing structures and can also be found in edentulous patients [1,2,3,4,5,6]. The most common periapical form is found first at the anterior mandibular molars, followed by the area of the mandibular angle. Usually, vital periapical tooth structures are embedded in COD; however, infections/inflammations might easily cause bone inflammation, namely osteomyelitis [8,9,10,11]. All possible solid, radiopaque lesions in the jaw bones require detailed diagnoses, especially the evaluation of the status of cortical bone, teeth mobility, and their reaction to cold stimuli, followed by any possible signs of bone inflammation or formation of other similar features [8,9,10,11,12]. The current article is a retrospective study of epidemiological, clinical, and radiological COD data found in the western part of Poland during the collaboration of our multidisciplinary team.

## 2. Material and Methods

### 2.1. Study Design

The following paper is a retrospective study based on a radiological database of patients treated by our multidisciplinary team between 2015–2023. The participants enrolled in the study were either consulted, treated, operated upon, or scheduled for various oral, maxillofacial, or ENT procedures, during which COD was found or diagnosed. Based on the radiological data, RTG-panx all of the participants were divided according to age and sex, and type, place, and side of occurrence of COD. The following radiographies were studied in adults > 18 years of age. These patients had CBCT and RTG-panx performed for various reasons, such as impacted teeth, jaw pain, cysts/tumors of the jaw, orthognathic surgery, condylar hyperplasia, evaluation of bone/mandible asymmetry, odontogenic sinusitis, bone inflammation, and other issues. Inclusion criteria for the study comprised data only from the authors’ database where patients are treated and diagnosed without any relevant medical history concerning any other pathologies in the craniofacial skeleton or their treatment. Exclusion criteria were as follows: other cystic and/or solid lesions without any features of COD, cases of past surgery in the area of any radiopaque lesions, lack of patient approval for the study, inadequate radiographies panx/CBCT without visible bone lesions.

The research respected the ethical principles of the Helsinki Declaration (2008) and the CBCT guidelines. For this research to begin, ethical approval was first signed by the Bioethics Committee nr 2-4-BNR-2022 and the newest nr 19/BOBD/2024 form 11 September 2024.

### 2.2. Radiography Characteristics

**Figure 1:**
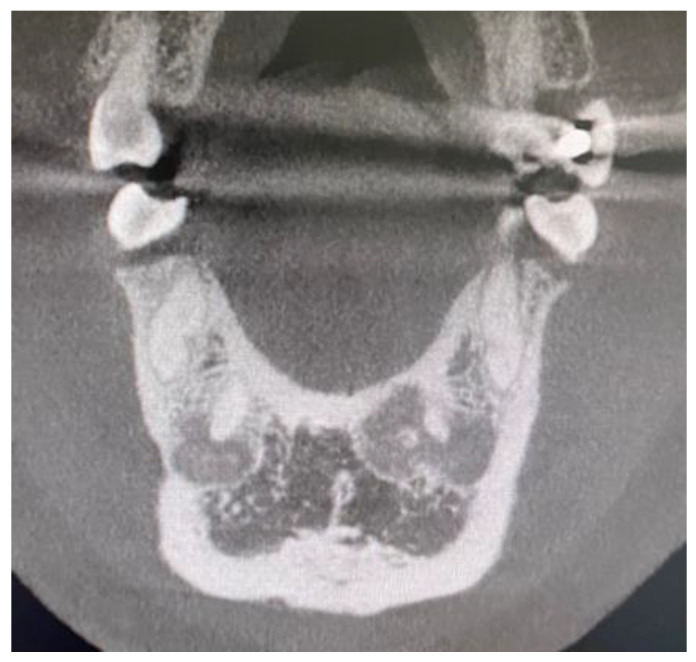
CBCT, coronal view, COD surrounding the periapical area of the mandibular incisors.

A detailed review of the clinical practice database between 2015–2023 was performed. All RTG-panx/CBCT diagnosed as COD from the authors’ database were studied in the RadiAnt Dicom Viewer Software (Medixant, Poznań, Poland). Radiological data was closely evaluated based on the bone characteristics and the radiopaque/radiolucent characteristics of each bone jaw lesion characteristic for COD (Figure 1). All gathered data and variables were categorized and put into each specific patient’s archive.

### 2.3. Methods—Radiological Measurements

**Figure 2:**
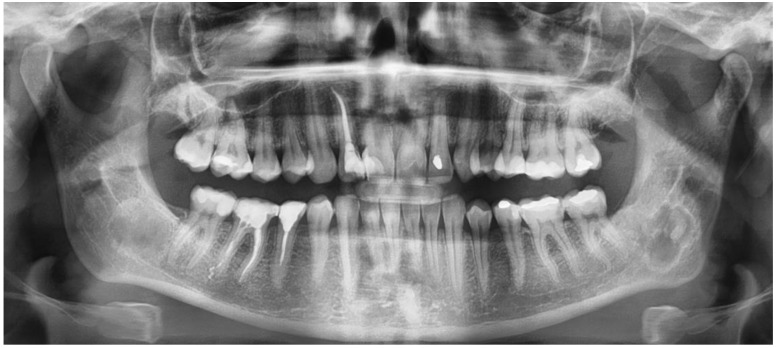
RTG-panx with visible COD at close proximity of both mandibular angles.

**Figure 3:**
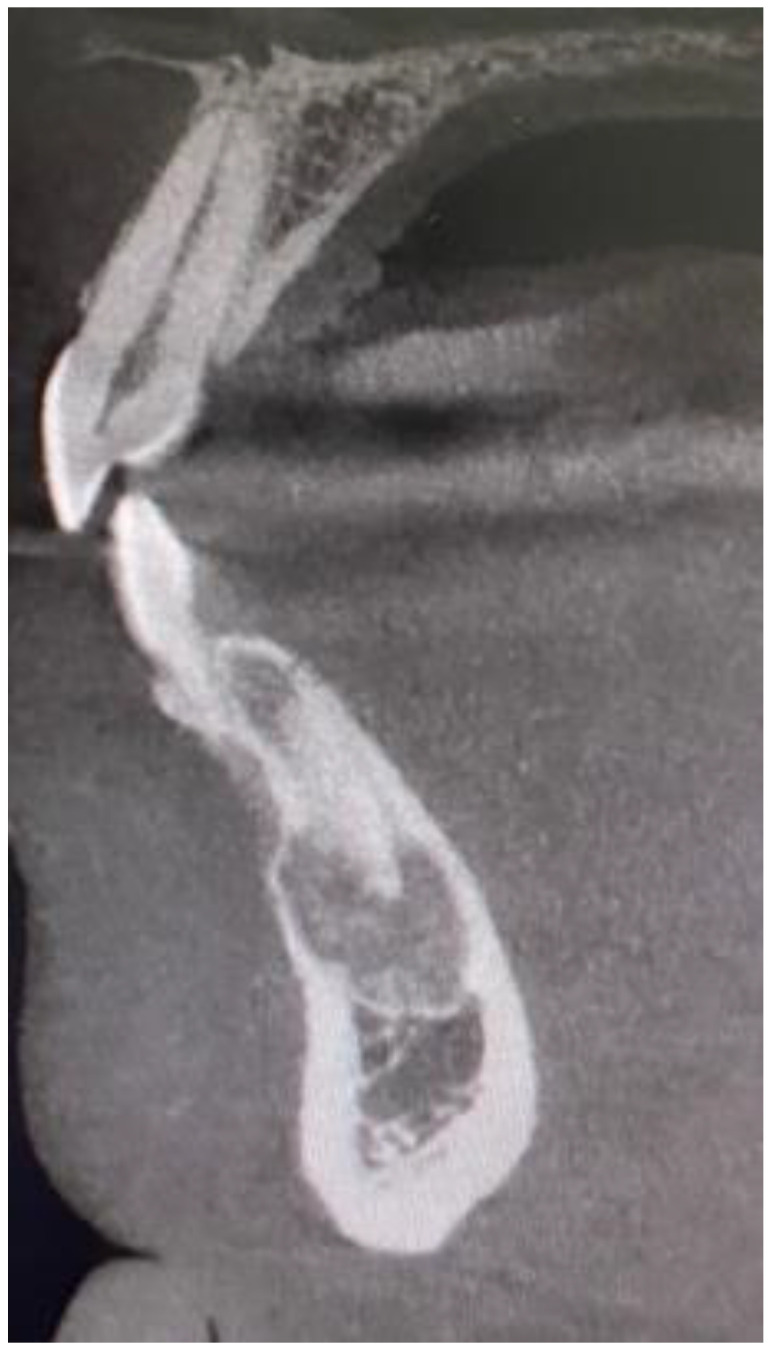
CBCT sagittal view of COD at the mandibular anterior symphysis region.

**Figure 4:**
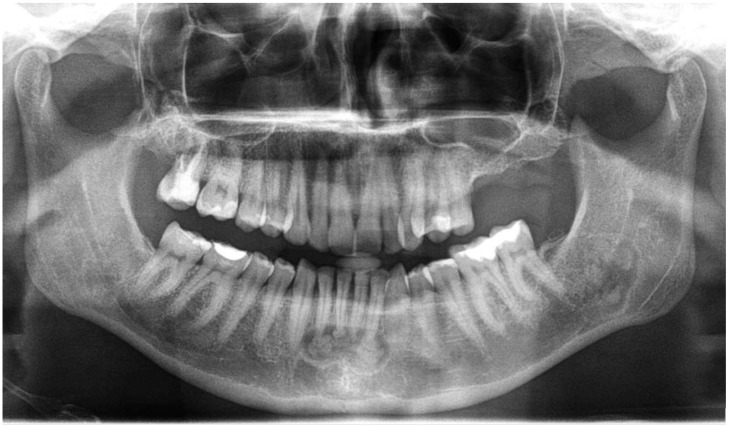
RTG-panx with visible COD at the anterior mandibular and left retromandibular areas.

**Figure 5:**
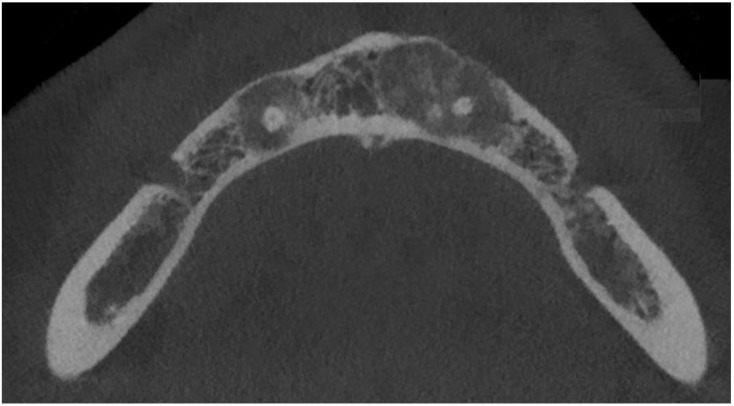
CBCT-axial view on COD in the anterior mandibular base. Most common occurrence side of COD.

**Figure 6:**
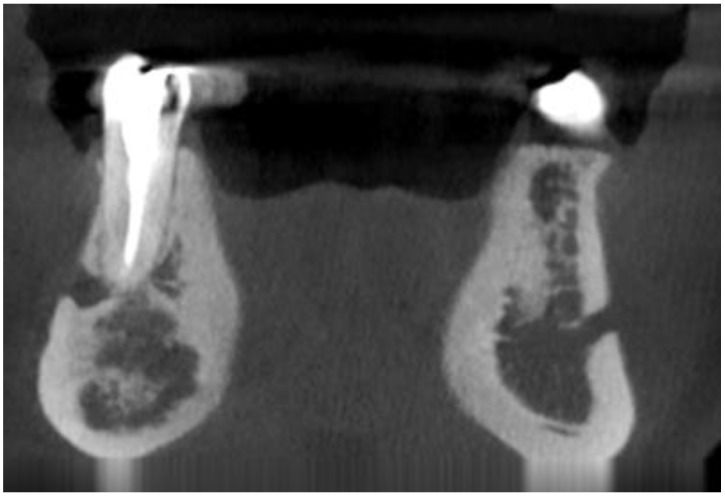
CBCT-coronal view, COD in the premolar area in the right mandibular basis.

**Figure 7:**
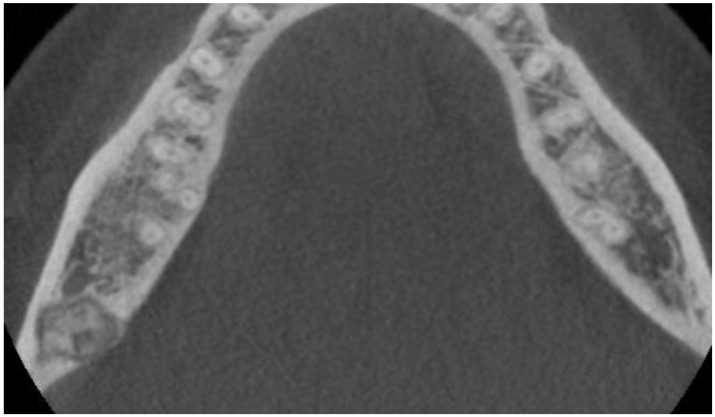
CBCT axial view with COD spread towards the lingual cortical plate.

**Figure 8:**
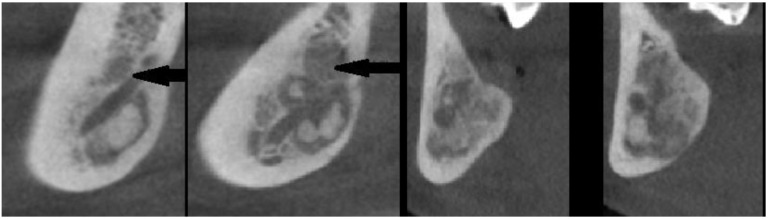
Sagittal CBCT projections of COD surrounding the inferior alveolar nerve without any visible compression or changes in the bony canal shape and position. Black arrow points on the mandibular canal and the lesion located within the bone canal.

The following lesions of the jaw bones were classified as COD in the radiological (CBCT/panx) evaluation (Figure 1, Figure 2, Figure 3, Figure 4, Figure 5, Figure 6, Figure 7 and Figure 8):

- mixed radiolucent/radiopaque lesion (depending on the stage of COD maturation), Figure 1.
- present in the jaw/maxilla in tooth-bearing areas, Figure 3
- radiolucent with/without radio-opacities with thin radiolucent rim, Figure 2
- self-limiting radiolucency, Figure 4
- associated with anterior/posterior teeth (Figure 2)
- various stages of calcifying masses inside of the lesion, Figure 5
- may cause cortical expansion or teeth displacement, Figure 5.
- no changes within the teeth root apex, Figure 3
- single, bilateral, or florid occurrence, Figure 1, Figure 2, Figure 3, Figure 4, Figure 5, Figure 6, Figure 7 and Figure 8.

All radiographies were gathered from author databases and then closely evaluated by the authors (K.N.), (J.N.).

### 2.4. Statistical Analysis

This study is primarily based on quantitative variables; thus a chi-squared test was employed. When the requisite conditions for a chi-squared test were not met, a Fisher’s exact test was utilized. In instances where statistical tests were not applicable, only descriptive statistics are presented. The statistical inference was supported by Jamovi 2.5 [13], which employed the R statistical environment [14].

## 3. Results

### 3.1. COD and Sex-Related Differencess

**Figure 9:**
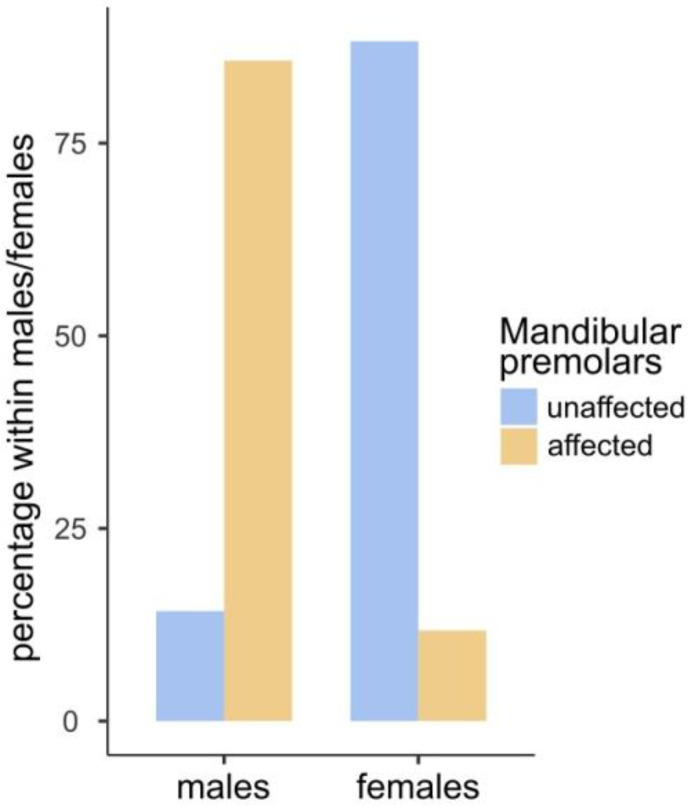
The bar plot illustrates the sole statistically significant difference between the sexes. Males exhibited a greater propensity for mandibular molar involvement than females. The Fisher’s exact test yielded a *p*-value of less than 0.001, indicating a statistically significant difference.

There were no statistically significant differences between males and females in the occurrence of COD in the maxilla or mandible. Furthermore, no correlation between age and COD occurrence was noted. It seems that COD has a totally random pattern of occurrence which is uniquely individual for each study participant, and COD localization was independent of sex (Figure 9). However, female patients were diagnosed six times more commonly with these bone lesions than men.

### 3.2. Multifocal and Single COD Localizations

**Figure 10:**
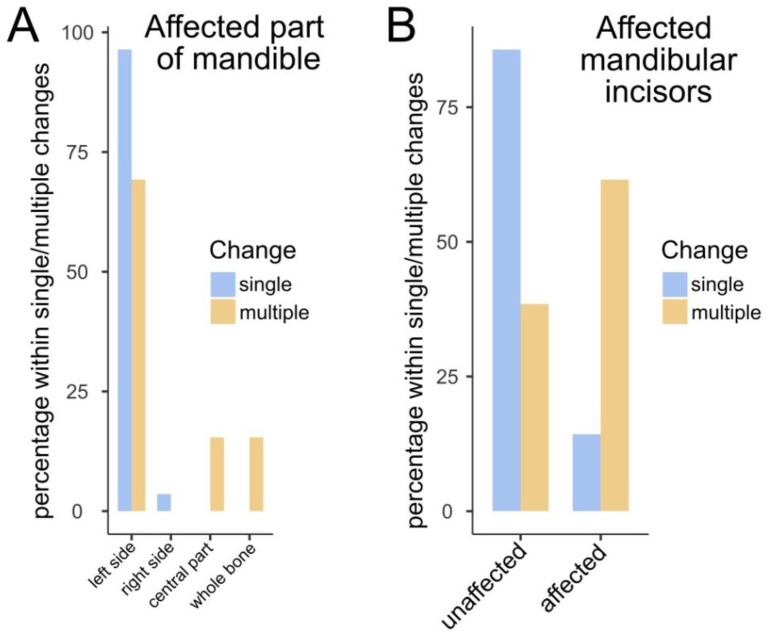
Bar plots illustrating the statistically significant findings pertaining to the occurrence of single or multiple changes in different locations. Plot (**A**) shows the occurrence of single/multiple CODs in the distinct regions of the mandible, and (**B**) shows the involvement of the mandibular incisor(s). The Fisher’s exact test yielded a *p*-value of 0.007 for data presented in plot (**A**) and 0.004 for plot (**B**).

Statistically significant relationships between COD localization and type (single/multiple) are shown in Figure 10. In the study population, single CODs in the mandible tended to be localized almost exclusively on the left side, whereas multifocal changes were also observed in the central part of the mandible and in the whole bone. Also, when the mandibular incisors were involved, COD tended to be multifocal rather than single.

### 3.3. COD Symptoms and Decision-Making

**Figure 11:**
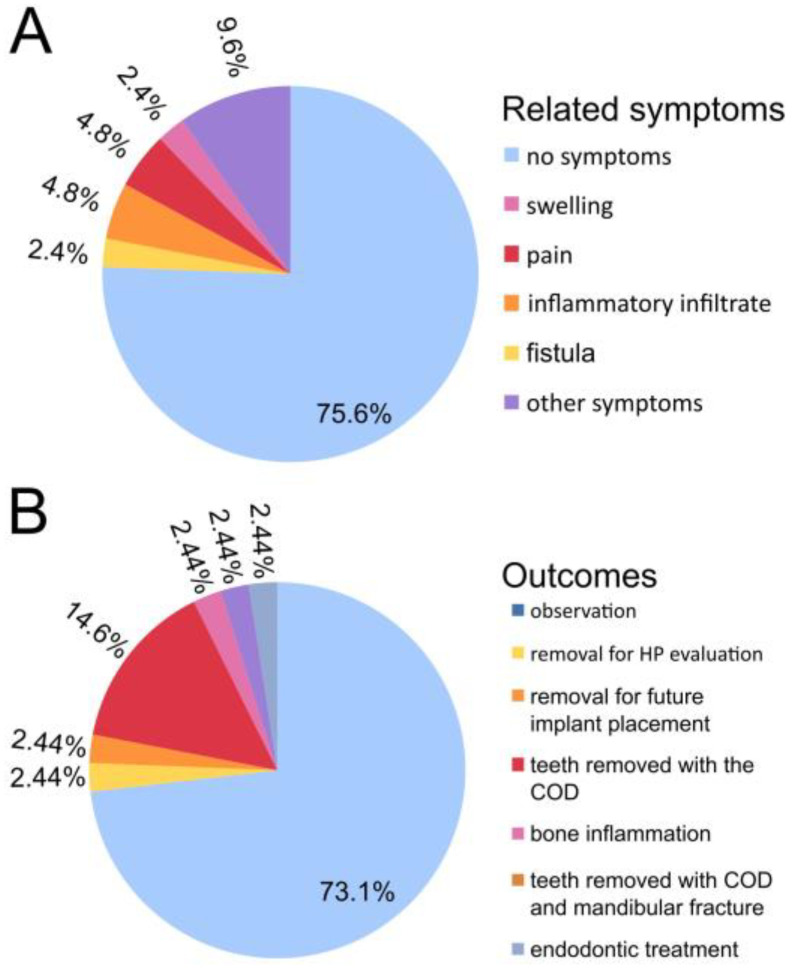
Pie chart illustrating the frequency of two categories: (**A**) different symptoms related to the COD and (**B**) outcomes.

Most often the CODs found were asymptomatic (75.6%). Swelling and pain occurred in less than five percent of cases; however some atypical, hard-to-describe symptoms near the bone and the COD lesion were atypical in ten percent of cases (Figure 11, Table 1). When swelling and pain was found at the COD lesion, this condition was mostly associated with inflammation and a nearby fistula. It is worth noting that CODs mostly remain asymptomatic, and any possible symptoms that may occur might be easily misdiagnosed with a tootchache.

### 3.4. COD Outcomes

COD occurrence and its symptoms greatly influenced the possible surgical approaches that were used. In most cases, if asymptomatic, only observation was advised (73.2%), except in one case where a tooth with COD was removed. In cases of a scheduled tooth removal or possible dental implant placement, the lesions were simultaneously removed. Rarely the COD occurrence was correlated with impacted teeth removal and possible mandibular fracture or the necessity of scheduled endodontic treatment within proximity of the COD (Table 2).

### 3.5. COD Localization and Symptoms

**Figure 12:**
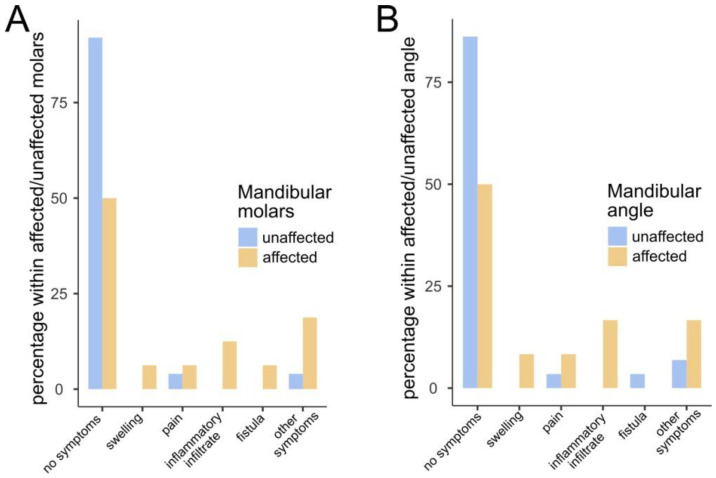
Bar plots for statistically significant results concerning the occurrence of distinct symptoms and (**A**) affected mandibular molars and (**B**) affected mandibular angle. When any of those sites was affected, COD was less likely to be asymptomatic. The Fisher’s exact test *p*-value for (**A**) is 0.008 and for (**B**) it is 0.025.

Figure 12 shows the statistically significant relationships between symptoms and locations affected by COD. In particular, when the mandibular molar of the mandibular angle was affected, COD tended to be less often asymptomatic. Similar relationships were not observed for different sites in the current study (Table 3 and Table 4). It looks like COD in single form is more common that the multifocal one, while its localization and symptoms are case dependent and not statistically significant (Table 5).

## 4. Discussion

Both osseous and fibro-osseous lesions of the jaw bones require good diagnostic skills. A detailed patient clinical evaluation should always be improved by additional CBCT evaluation. The large amount of different pathologies that can be found in CBCT and RTG-panx require skill and knowledge among dentists, surgeons, and clinicians of other different pathologies. Alsufyani and Lam conducted a study on possible diagnostic differences in radiological skills between general dentists and maxillofacial radiologists in COD identification and interpretation and concluded that only more qualified radiologists were more likely to identify these lesions (79.3% vs. 38.7%) [11]. It is also worth pointing out that unnecessary endodontic treatment is sometimes scheduled, as are biopsies or tooth removal because of COD.

The occurrence of COD is not common [1,2,3,4,5,6,7,8]. The majority of authors report a predominance in females over males [5,6,7,8,9,10,11,12,13,14,15]. It is also quite important to note that the following study is the first study from western Poland, and it is a very important study to improve recent literature on COD occurrence. Furthermore, Polish geographical data also confirm that more females are diagnosed with COD.

The appearance of most fibro-osseous lesions in jaw bones might be troublesome to differentiate. Both sclerotic, fibrous cemento-fibrous, osseo-fibrous, or related pathologies in jaw bones might have similar histopathologic features. Because of this, the radiological and clinical symptoms (e.g., tooth pulp vitality, teeth mobility, pain, and swelling) might have an influence on a subsequent final diagnosis of the lesion found in the jaw bone. CODs are a type of varied fibro-osseous lesions that might occur in jaw bones in close relation to the alveolar part of both the mandible and the maxillary bones [9,10,11,12]. It is worth pointing out that COD can be divided into periapical, florid, and similar subdivisions, all of which can be found on radiographies during various stages of growth, maturation, and formation, which may lead to misdiagnoses. The most common ones include the misinterpretation of radiographic imaging with periapical granuloma, radicular cyst, radicular abscess, periodontal cyst, periapical osteitis, idiopathic osteosclerosis, or similar purely odontogenic-like features [5,7,9,10,11]. Those features might correlate with the bone degree of maturation, mixed radiolucent–radiopaque areas, cortical bone involvement, and the condition of the periodontal space.

Salvi et al.’s study concludes that COD is asymptomatic; however, it rarely progresses towards florid COD [9]. The degree of radiopacities, calcifications, possible cortical expansions, peripheral bone visualization (radiolucent bend/rim), and other features might vary greatly. Some authors conclude that COD should also be differentiated from some jaw bone inflammations or inflammation-like processes, furthermore, COD can quite easily promote local inflammation if odontogenic infection or inflammation is present [2,3,4,5,6,7,8]. Benaessa et al.’s study points out that COD-related jaw osteomyelitis might be related to the status of dental hygiene and general teeth condition among the studied African population [10].

On the other hand, in the authors’ study just one patient had jaw osteomyelitis with purulent fistulas related to poor oral hygiene and secondary COD inflammation [10,11,12]. It seems that maintaining good oral hygiene and a good condition of the vital teeth is the most important aspect when a COD lesion is diagnosed. In the authors’ study, the occurrence of inflammation, purulent fistulas, and related features was rare; however, some atypical and troublesome features were also randomly present. To summarize, only asymptomatic COD lesions or those COD lesions that would affect dental implant placement or teeth removal required surgery. In these cases a biopsy was performed to fully evaluate the scope of the lesion [12,13,14,15].

It seems that routine panoramic radiographies are not sufficient in all cases to distinguish COD from other osseous, fibro-osseus or bone lesions in the jaw bones. A good diagnostic study was presented by Kato et al. [15,16,17]: the authors evaluated radio-morphometric parameters of mandibular, trabecular, and cortical bones of females with and without cemento-osseous dysplasia on panoramic radiographies. The outcomes of the study highlight that females with COD had lower values in mandibular cortical bone structure than those without COD, without any changes in the alveolar trabecular bone. Kato et al. indicate that not only is good dental and oral hygiene screening necessary, but routine evaluation of low bone mineral density should also be considered [17,18,19]. It is not yet confirmed if osteopenia or osteocytopenia patients’ bone characteristics for different ages and sexes might correspond with an early or late occurrence of COD. It is worth establishing some geographical differences in COD occurrence, where single COD was less symptomatic, compared to some multiple/florid types (FLCOD) [17,18,19,20]. Decolibus et al.’s study, as well as other authors, suggest that COD is quite often found accidentally in middle-aged females of African descent and occurs more frequently in the mandible, with more female predominance; however, some Eastern Asia pattern occurrence was also noted [10,12,13,14,15,16,17,18,19,20,21,22].

Because COD might be found as a single, focal, or multifocal lesion in the jaw bone, some special attention should be established, not only to evaluate the type of bony lesion but also to either confirm or deny a possible coincidence with other jaw bone lesions. COD is characteristic for the preservation of the periodontal ligament, which is usually intact and does not resorb [12,13,14,15,16,17,18,19,20]. Yeom and Yoon’s study described a simultaneous occurrence of COD and ABC (aneurysmal bone cyst) [16]. It seems that both cysts and lesions with or without epithelial lining could be also found in the jaw bone alongside COD. On the other hand, when evaluating any radiographies or CBCTs it is worth noting that COD can exhibit radiological, clinical, and histopathological features similar to other bone fibro-osseous lesions; this is also confirmed by Olgac et al. [17]. While a mandible with mixed radiopaque–radiolucent radiographic features is the most common feature, other similar radiological features can also be found, like odontogenic and non-odontogenic cysts [10,11,12,13,14,15,16,17,18]. Differential diagnoses in CBCT, patient follow-up, and both clinical and radiological COD evaluation are essential because proper control is always better than unnecessary surgical intervention. Each bone lesion growth, tooth apex resorption, and change in shape, size, and volume of the lesion with teeth malpositioning should correlate with the necessity of a biopsy. In some troublesome cases, either an improved diagnosis, a comparison of old radiographies, or, in some cases, a biopsy with bone revision might influence the final diagnosis. Because the radiological appearance can mimic fibro-osseous lesions, during radiographic evaluation, the occurrence of some coexisting lesions might be found. In the authors’ paper there were no signs of any other cysts, lesions, or bone pathologies within the evaluated material.

Because of new technologies in dentistry and radiology, such as CBCT and other diagnostic tools, it is quite possible to establish many bone features. The first and most important ones are the shape, contour, symmetry, and size of each jaw bone, especially the mandible. Additional features can help in establishing the thickness of cortical bone, the degree and volume of trabecular bone, and the correlation between each jaw bone lesion and the bone condition itself. A study by Arsan with CBCT evaluated the cortical thickness in COD patients and concluded that female patients had decreased cortical volume compared to the healthy bone-lesion-free control group [19].

Some considerations for future perspectives include the necessity of correlating the occurrence of any co-existing endocrine diseases, skeletal abnormalities, and other bone, osseous, and fibro-osseous lesions with bone markers, and, perhaps in the future, investigating how COD is forming in the jaw bones. The cortical bone condition and occurrence of COD might have an impact on future dental placement in edentulous patients as reported by Mlouka et al. [20]. Furthermore, any possible symptomatic cases with inflammation of bone with COD might not only have an impact on future dental implant placement but also might constrain other bone surgery to remove the inflammed bone, which might greatly impact future bone condition when dental implant rehabilitation is considered [21,23]. Some studies indicate that bone inflammation causing osteomyelitis (OM) is more often found in COD patients than in any other group [10,22]. On the other hand, the presence of teeth embedded with COD should require a differential diagnosis with other cysts and tumor-like pathologies related to impacted, embedded, or partially retained teeth [10,11,12,20,21,22,23]. Any biopsy attempt on CODs and related lesions might influence the occurrence of possible jaw bone inflammation. In each case, a detailed differential diagnosis is mandatory before any biopsy is scheduled; it is especially worth knowing what other diseases in the periodontal area or other atypical co-existing morbidities can be found simultaneously [24,25,26,27,28,29].

Study limitations include:—number of patients diagnosed with COD;—a great variety of shapes and sizes of COD and their relation with tooth roots;—missing adequate CBCT imaging when only a panoramic radiography is available;—limited histopathological studies since most COD lesions were controlled only in radiographies;—missing data and cases of coexistence of COD with other cysts, tumors or lytic lesions in the jaw bones;—impossible to estimate status of cortical bone because of a great variety of COD shapes and sizes;—missing long-term data on possible COD growth, maturation, and calcification stages over the years.

## 5. Conclusions

Most COD bone lesions are found accidentally in the mandibular bone. Quite often they are asymptomatic, with various shapes, sizes, and locations. COD seems to be more common in females; however, no age and side of occurrence relations were found. It is worth concluding that only symptomatic COD lesions require any surgical intervention. CODs can be easily diagnosed on CBCT and panx, and good oral hygiene is important to avoid any COD-related inflammation occurrence.

## Figures and Tables

**Table 1:** Descriptive statistics on the symptoms related to COD.

Symptom	Count	Proportion
no symptoms	31	75.6%
swelling	1	2.4%
pain	2	4.9%
inflammatory infiltrate	2	4.9%
fistula	1	2.4%
other symptoms	4	9.8%

**Table 2:** Descriptive statistics on the outcomes related to COD.

Outcome	Count	Proportion
observation	30	73.2%
removal for HP evaluation	1	2.4%
removal for future implant placement	1	2.4%
teeth removed with the COD	6	14.6%
bone inflammation	1	2.4%
teeth removed with COD and mandibular fracture	1	2.4%
endodontic treatment	1	2.4%

**Table 3:** Contingency tables for differences between sexes in relation to affected/unaffected premolars. Fishers exact test *p*-value < 0.001.

		Mandibular Premolars		
Sex		Unaffected	Affected	Total
Male	Observed	1	6	7
	Expected	5.29	1.71	7
Female	Observed	30	4	34
	Expected	25.71	8.29	34

**Table 4:** Contingency table for COD-related symptoms and outcomes. No symptoms in most cases (30/31) resulted only in observation. Fishers exact test *p*-value < 0.001.

		Outcome						
		Observation	Removal for HP Evaluation	Removal for Future Implant Placement	Teeth Removed with the COD	Bone Inflammation	Teeth Removed with COD and Mandibular Fracture	Endodontic Treatment
no symptoms	Observed	30	0	0	1	0	0	0
	Expected	22.68	0.76	0.76	4.54	0.76	0.76	0.76
swelling	Observed	0	0	0	1	0	0	0
	Expected	0.73	0.02	0.02	0.15	0.02	0.02	0.02
pain	Observed	0	0	0	1	0	0	1
	Expected	1.46	0.05	0.05	0.29	0.05	0.05	0.05
inflammatory infiltrate	Observed	0	0	0	1	1	0	0
	Expected	1.46	0.05	0.05	0.29	0.05	0.05	0.05
fistula	Observed	0	0	0	1	0	0	0
	Expected	0.73	0.02	0.02	0.15	0.02	0.02	0.02
other symptoms	Observed	0	1	1	1	0	1	0
	Expected	2.93	0.10	0.10	0.59	0.10	0.10	0.10

Abbreviations: COD, cemento-osseous dysplasia.

**Table 5:** Final comparison of study data.

Sex		n
	Males	7
	Female	34
Mean age	36.7 (SD = 9.36)	
Affected bone:		
	Upper jaw	5
	Lower Jaw	40
Affected teeth/site:		
	Maxillary incisors	2
	Mandibular incisors	12
	Mandibular canine	11
	Mandibular premolars	10
	Mandibular molars	16
	Mandibular angle	12
	Mandibular symphysis	4
Multiplicity		
	Single	28
	Multifocal	13

Abbreviations: SD—standard deviation, n—number.

## Data Availability

Availability of supporting data—The datasets used and/or analyzed during the current study are available from the corresponding author on reasonable request.
